# Plasma Nitration of High-Density and Low-Density Lipoproteins in Chronic Kidney Disease Patients Receiving Kidney Transplants

**DOI:** 10.1155/2015/352356

**Published:** 2015-11-15

**Authors:** Ahmed Bakillah, Fasika Tedla, Isabelle Ayoub, Devon John, Allen J. Norin, M. Mahmood Hussain, Clinton Brown

**Affiliations:** ^1^Department of Cell Biology, SUNY Downstate Medical Center, Brooklyn, NY, USA; ^2^The Department of Veterans Affairs New York Harbor Healthcare System, Brooklyn, NY, USA; ^3^Department of Medicine, SUNY Downstate Medical Center, Brooklyn, NY, USA; ^4^Department of Surgery, SUNY Downstate Medical Center, Brooklyn, NY, USA

## Abstract

*Background*. Functional abnormalities of high-density lipoprotein (HDL) could contribute to cardiovascular disease in chronic kidney disease patients. We measured a validated marker of HDL dysfunction, nitrated apolipoprotein A-I, in kidney transplant recipients to test the hypothesis that a functioning kidney transplant reduces serum nitrated apoA-I concentrations. *Methods*. Concentrations of nitrated apoA-I and apoB were measured using indirect sandwich ELISA assays on sera collected from each transplant subject before transplantation and at 1, 3, and 12 months after transplantation. Patients were excluded if they have history of diabetes, treatment with lipid-lowering medications or HIV protease inhibitors, prednisone dose > 15 mg/day, nephrotic range proteinuria, serum creatinine > 1.5 mg/dL, or active inflammatory disease. Sera from 18 transplanted patients were analyzed. Four subjects were excluded due to insufficient data. Twelve and eight patients had creatinine < 1.5 mg/dL at 3 and 12 months after transplantation, respectively. *Results*. Nitrated apoA-I was significantly reduced at 12 months after transplantation (*p* = 0.039). The decrease in apoA-I nitration was associated with significant reduction in myeloperoxidase (MPO) activity (*p* = 0.047). In contrast to apoA-I, nitrated apoB was not affected after kidney transplantation. *Conclusions*. Patients with well-functioning grafts had significant reduction in nitrated apoA-I 12 months after kidney transplantation. Further studies are needed in a large cohort to determine if nitrated apoA-I can be used as a valuable marker for cardiovascular risk stratification in chronic kidney disease.

## 1. Background

Cardiovascular disease (CVD) is the leading cause of death among patients with end-stage renal disease (ESRD), accounting for approximately half of all deaths [1–4]. The major course of treatment is dialysis. Dialysis patients die at six to seven times the rate of individuals in the general population with otherwise similar risk factors. Patients with less severe stages of chronic kidney disease (CKD) also carry excess risk of cardiovascular mortality that is not explained by traditional risk factors [5, 6]. Reduction of LDL cholesterol using statins lowers risk of atherosclerotic events in nondialysis CKD patients [7, 8]. However, similar protection by statins was not observed in clinical trials involving patients on dialysis [9, 10].

It is possible that HDL's quantity and quality may explain some of the residual risks in patients with advanced CKD. Epidemiologic studies have shown that there is a gradual increase in the risk of CVD as HDL cholesterol decreases [11, 12]. However, recent pharmacologic interventions aimed at increasing HDL abundance failed to provide clinical benefits and were associated with unexpected side effects despite promising preclinical outcomes [13, 14].

Recent studies are now focusing on assessing HDL function rather than measuring its traditional plasma concentration [15–17]. In this regard, attempts have been made to measure nitrated apoA-I. These studies have advanced the concept that oxidative modification of HDL by MPO renders HDL dysfunctional [18–20]. Elevations in nitrated apoA-I containing HDL have been reported in atherosclerotic plaques and in plasma of CVD patients [6, 21–23].

Both qualitative and quantitative changes in HDL have been described in patients with CKD [24]. Specifically, HDL abundance is reduced and HDL acquires a proinflammatory property instead of its usual anti-inflammatory role [25, 26]. In addition, HDL from patients with CKD has reduced capacity for reverse cholesterol transport [27]. In more recent study, CKD has been shown to alter specific HDL functions linked to control of inflammation and endothelial responses [28]. Low HDL levels were associated with earlier entry in dialysis or doubling of plasma creatinine levels independently of the presence of diabetes [29]. Immunohistochemistry staining revealed higher nitrotyrosine in arteries with media calcification in CKD patients [30]. Moreover, plasma proteins of CKD patients showed a higher burden of nitration than that in healthy controls [31]. Despite these significant reports on plasma nitroproteome, the implication of nitrated lipoproteins in disease progression has not been explored in CKD patients.

Degree of nitration in apoA1-HDL has been evaluated by several methods such as mass spectrometry and Western blot analysis in different disease setting [23, 32, 33]. However, all these techniques are time-consuming and are not well adapted to high throughput screen setting. We have developed an ELISA based method to quantify serum nitrated apoA-I. We showed that CVD subjects have low total HDL but their nitrated apoA-I content is high [34]. Using the same ELISA, Vazquez et al. demonstrated a significant decrease in cholesterol efflux by ABCA1 transporters and impaired endothelial function that were associated with increased nitration of apoA-I-HDL in obese women [35]. Patients with CKD are at increased cardiovascular risk and have reduced HDL levels and altered HDL composition [36]. We have therefore investigated whether levels of circulating nitrated lipoproteins change in CKD patients treated with kidney transplantation.

## 2. Subjects and Methods

### 2.1. Patients Selection and Clinical Variable Assessment

Seventy-eight subjects were recruited from outpatient clinics of SUNY Downstate Medical Center between November 2010 and June 2013. The study was approved by the Institutional Review Board under protocol number 441318-1. Written informed consent was obtained from all study participants. All participants were adults. Kidney transplant recipients were enrolled in the study unless they met exclusion criteria. Patients with the following criteria were excluded from the study: diabetes, HIV on antiretroviral therapy, active systemic rheumatologic diseases, nephrotic syndrome, protein : creatinine ratio >1, treatment with lipid-lowering agents, body mass index (BMI) > 35, creatinine >1.5 mg/dL or estimated glomerular filtration (eGFR) <60 mL/min by Modification of Diet for Renal Disease (MDRD) equation, steroid dose >15 mg prednisone or equivalent a month or more after transplantation, and treatment with sirolimus or everolimus. All these above selection criteria would eliminate most of important confounding factors that could interfere with lipid and lipoprotein metabolism. A sample size of 12 was estimated to provide 90% power to detect difference of 448 *μ*g/dL between pretransplant and 12-month posttransplant concentration of nitrated apoA-I at two-tailed type I error *α* of 0.05 using Wilcoxon signed-rank test [34, 35].

Sera were collected from each transplant subject before transplantation and at 1, 3, and 12 months after transplantation. In total, sera from eighteen transplanted patients who were eligible and presented stable kidney function were retrospectively analyzed. Four patients were excluded due to incomplete time point data. At the end, among this group, twelve and eight patients had creatinine < 1.5 mg/dL at 3 and 12 months after transplantation, respectively.

### 2.2. Sample Preparation and Quantification of Nitrated Lipoproteins by ELISA

Aliquoted sera stored at −70°C were thawed and concentrations of nitrated apoA1-containing HDL were measured using a well-established sandwich ELISA method [34]. A similar sandwich ELISA has been developed to measure levels of nitrated apoB-containing LDL particles as well. The specificity of the ELISA assay was described previously [34]. The intra-assay and interassay coefficients of variation for nitrated lipoproteins and apolipoproteins measurement were less than 5% and 10%, respectively. These values are consistent with the precision of typical sandwich ELISA assays [34, 37, 38]. Briefly, diluted sera samples were incubated in 96-well plates previously coated with monoclonal anti-nitrotyrosine antibodies (EMD Millipore, Billerica, Ma, USA; clone 1A6, Cat.# 05-233), enabling the capture of total nitrated serum proteins including apoA-I and apoB. The plates were blocked in PBS buffer containing 3% bovine serum albumin (BSA) and washed with PBS-Tween (0.05%). Primary polyclonal antibodies to human apoA-I or apoB (Novus Biologicals, Littleton, CO, USA; Cat.# NB400-147 and Cat.# NB120-7616, resp.) were added to specifically bind nitrated apoA-I or nitrated apoB captured by the anti-nitrotyrosine antibodies. Standard curves were generated using increasing concentrations (1–100 ng/mL and 1–100 *μ*g/mL) of purified human serum HDL and LDL, respectively (MyBioSource, San Diego, CA, USA; Cat.# MBS173145 and Cat.# 173147). For this purpose, monoclonal antibodies against apoA-I and apoB were immobilized in 96-well plates (4H1 and 1D1, resp., University of Ottawa Institute, Ottawa, Canada). Bound HDL (apoA-I-HDL) and LDL (apoB-LDL) were detected with alkaline phosphatase-conjugated secondary antibodies and p-Nitrophenyl phosphate (pNPP) as substrate (1 mg/mL).

Absorbance at 405 nm was measured and corrected to absorbance of control wells in which PBS-Tween was added instead of serum. Total serum apoA-I and apoB levels were determined as described previously [34, 35]. Absolute values of nitrated apolipoproteins (apoA-I and apoB) were normalized to total serum apoproteins and degree of nitration was expressed as percent.

### 2.3. Lipid Analyses and Enzymatic Assays

Non-HDL fraction was isolated from sera by precipitating (LDL/VLDL) with manganese chloride solution (1.06 M). After centrifugation, total cholesterol in the supernatants (HDLc) and in PBS-reconstituted precipitates (non-HDLc) was measured by colorimetric assay (Wako Diagnostics, Richmond, VA, USA; Cat.# 439-17501). Lipid peroxidation/oxidative stress were evaluated by measuring thiobarbituric acid reactive substances (TBARS) in sera. Briefly, the LDL/VLDL fraction was precipitated by 1.06 M manganese chloride and levels of TBARS were determined using OXI-TEK TBARS assay kit (ZeptoMetrix Corp., Buffalo, NY, USA; Cat.# 0801192). MPO was measured by colorimetric activity assay kit (Sigma, St. Louis, MO, USA; Cat.# MAK068).

### 2.4. Statistical Analysis

Only results for those with creatinine < 1.5 (those with successful transplant) are mentioned. Data for continuous variables (means ± SD) and medians (interquartile ranges) were reported. All analyses were performed using the Prism GraphPad 5.0 and Statistica 10.0 softwares. Paired values of percent nitrated apoA-I or nitrated apoB before and after transplantation were compared using nonparametric Wilcoxon signed-rank sum test. In addition, linear regression and Pearson's correlation coefficient were used to assess associations between variables. *p* < 0.05 was considered statistically significant.

## 3. Results

### 3.1. Quantification of Nitrated HDL and LDL by ELISA

**Figure 1:**
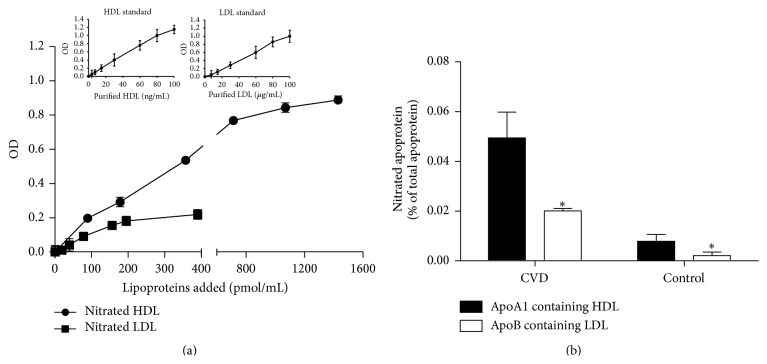
Panel (a) shows changes in optical densities due to the presence of nitrated apoA-I and apoB in the serum when immobilized anti-nitrotyrosine antibodies are incubated with increasing amounts of purified HDL and LDL. The amount of nitrated apolipoproteins present in the serum as measured by ELISA using commercially available purified human HDL and LDL as standard curves. Comparison between nitrated HDL (apoA-I-HDL) and nitrated LDL (apoB-HDL) was carried out using equimolar concentrations of HDL and LDL as described in methods. Representative linear standard curves for ELISA are plotted at the top of panel (a). Values are mean of triplicates ± SD. Panel (b) shows a comparison between percent nitration of apoA-I-HDL and apoB-LDL in human serum. Sera obtained from commercially available blood donors (Bioreclamation, LLC) were used. Mean values in these sera for apoB were 149.69 ± 28.78 mg/dL (range: 114.65−204.62 mg/dL) and for apoA-I were 49.30 ± 12.96 mg/dL (range: 35.06−73.35 mg/dL). Sera were distributed in two groups (control versus CVD; *N* = 10/group) based on their lipids and lipoproteins levels. Concentrations of nitrated apoA-I and nitrated apoB and lipoproteins levels were measured by ELISA. Final values of nitrated apoA-I and apoB were normalized by levels of HDL and LDL, respectively. Values are mean ± SD (*N* = 10/group). One-way ANOVA test was performed between the two groups. Statistical significance was considered at *p* < 0.05.

Figure 1(a) illustrates kinetic curve of detection of immobilized nitrotyrosine-bound lipoproteins in the wells by polyclonal antibodies against apoA-I and apoB. At equimolar concentrations, nitrated LDL binding reached saturation quicker than nitrated HDL, and the assay achieved linearity between 0 and 100 *μ*g/mL LDL added and between 0 and 100 ng/mL HDL added (top 2 panels in Figure 1(a)). Sera from CVD patients showed a twofold increase of nitrated lipoproteins levels as compared to healthy subjects. There was approximately a sixfold increase of nitrated molecules of HDL compared to LDL (Figure 1(b)).

### 3.2. Clinical Characteristics of Study Cohort

Patients meeting eligibility criteria as described above were analyzed in this study. Among the 18 transplanted patients who were eligible and presented stable kidney function four patients were excluded due to incomplete data and twelve and eight patients had creatinine < 1.5 mg/dL at 3 and 12 months after transplantation, respectively.

Subjects' age in this cohort ranged from 29 to 64 years. Clinical characteristics are summarized in Table 1. At baseline, the mean HDL cholesterol and the mean non-HDL cholesterol were 52.9 ± 16.7 mg/dL (22.4–85.3) and 107.1 ± 22.2 mg/dL (71.7–147.3), respectively. Mean value of triglycerides levels was 157.8 ± 89.6 mg/dL and apoA-I and apoB levels were 74.1 ± 12.4 mg/dL and 123.3 ± 29.8 mg/dL, respectively. Creatinine (Cr) levels ranged from 4.6 mg/dL to 13.2 mg/dL and C-reactive protein (CRP) levels were below 4 mg/L. There was a reduction in serum creatinine and there was a slight increase in BMI and hemoglobin levels 12 months after transplantation as compared to values at baseline.

### 3.3. Temporal Changes in Serum Components and Status of Nitrated Lipoproteins in the Total CKD Cohort

**Figure 2:**
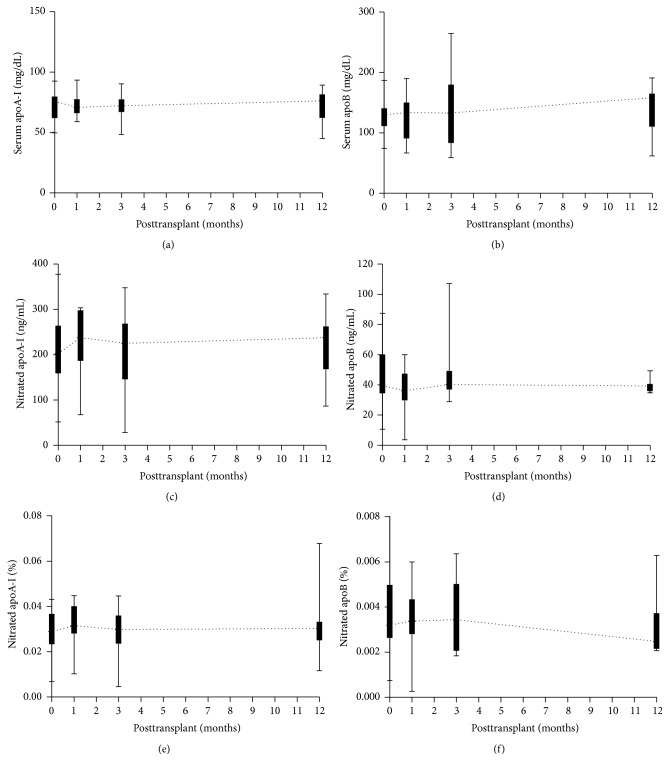
Temporal changes of circulating apolipoproteins and nitrated apolipoproteins in the 14 transplant patients with good kidney function before transplantation (baseline) and 1 month, 3 months, and 12 months after transplantation. Concentrations of total serum apoA-I containing HDL and apoB containing LDL particles (a and b) and levels of nitrated apoA-I and apoB (c and d) were measured by ELISA. Percentages of nitrated apoA-I and nitrated apoB were calculated by normalizing absolute values by total amount of apoA-I and apoB, respectively (e and f). Data are represented as box-and-whisker plots. Median values from each time point are connected to generate curves.

Overall, serum components such as total apoA-I and apoB did not change over time after transplantation (Figures 2(a) and 2(b)). Analysis of all fourteen patients with good graft function did not show any significant changes in nitrated lipoproteins 1 month, 3 months, and 12 months after transplantation (Figures 2(c)–2(f)).

### 3.4. Changes in Nitrated HDL and LDL in Kidney Transplant Recipients with Creatinine < 1.5 mg/dL

**Figure 3:**
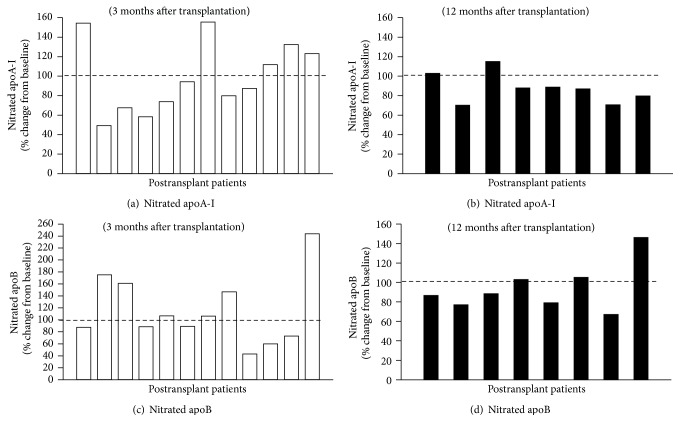
Percentage changes of nitrated lipoproteins (apoA-I-HDL and apoB-LDL) 3 months and 12 months after transplantation. Sera of twelve and eight patients with serum creatinine <1.5 mg/dL at baseline (before transplantation) and at 3 and 12 months after transplantation were analyzed by ELISA. Data represented are percent changes of nitrated apoA-I (a and b) and nitrated apoB levels (c and d) at 3 months and 12 months after transplantation, respectively. Baseline value for each patient was set at 100%. Paired values of percent nitrated apoA-I or nitrated apoB before and after transplantation were compared using nonparametric Wilcoxon signed-rank sum test. Statistical significance was considered at *p* < 0.05.

At the end of the study we had twelve patients and eight patients with good graft function (creatinine < 1.5 mg/dL) at 3 months and 12 months after transplantation, respectively. For each subject, we compared paired values of percent nitrated apoA-I and apoB before kidney transplantation and 3 months and 12 months after kidney transplantation. Analysis of the twelve patients that had creatinine < 1.5 mg/dL at 3 months after transplantation showed no difference in nitrated apoA-I (Figure 3(a)). At 12 months after transplantation, levels of apoA-I-HDL were slightly increased but did not reach significance (mean values were 73.4 ± 14.1 mg/dL and 76.7 ± 13.7 mg/dL at baseline and at 12 months after transplantation, resp.). This modest elevation of serum apoA-I levels was associated with significant reduction (~12%) in nitrated apoA-I (Figure 3(b)). Interestingly, six patients among eight have decreased their nitrated apoA-I by ~10%–30% 12 month after transplantation. The mean value for percent of nitrated apoA-I was significantly reduced by 18.5 ng/mg apoA-I (median value reduced by 22.5 ng/mg apoA-I; *p* = 0.039) 12 months after transplantation. In contrast, there were no significant changes in nitrated apoB at 3 months and 12 months after transplantation (Figures 3(c) and 3(d)). Like apoA-I, serum apoB levels tended to slightly increase but did not reach any significance (mean values were 112.46 ± 46.92 mg/dL and 141.65 ± 41.38 mg/dL at baseline and at 12 months after transplantation).

### 3.5. MPO Activity and Lipid Peroxidation

The decrease in apoA-I nitration 12 months after transplantation was associated with a significant reduction of MPO activity levels (Table 1; median values: 68.3 mU/mL versus 107.1 mU/mL; 32% decrease; *p* = 0.047). Lipid peroxidation as measured by TBARS assay was unchanged at 3 months and 12 months after transplantation.

### 3.6. Correlation between HDL and LDL Levels and Percent Nitrated apoA-I and Nitrated apoB

**Figure 4:**
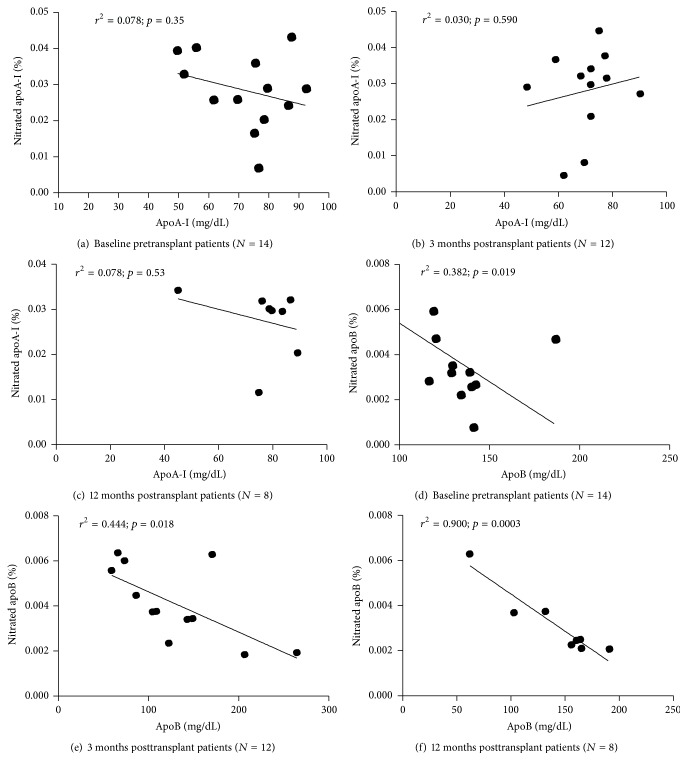
Correlations between % nitrated apoA-I and apoA-I-HDL levels and % nitrated apoB and apoB-LDL levels at baseline (before transplantation; a and d) and at 3 months (b and e) and 12 months (c and f) after transplantation, respectively. Linear regression was used to generate the curves. Statistical significance was considered at *p* < 0.05.

In our previous study, we have reported a negative relationship between degree of apoA-I-HDL and levels of circulating HDL particles in low HDL patients [34]. In this study, we sought to determine patterns of potential relationship between nitrated apolipoproteins and circulating lipoproteins in kidney transplant patients at baseline and at 3 months and 12 months after transplantation (Figure 4). There was no significant correlation between percent nitrated apoA-I and serum apoA-I levels in CKD patients before kidney transplantation (Figure 4(a)) and 3 months (Figure 4(b)) and 12 months (Figure 4(c)) after kidney transplantation. In contrast, there was a significant negative relationship between percent nitrated apoB and serum apoB levels at baseline (Figure 4(d)). This inverse correlation was maintained after 3 months (Figure 4(e)) and becomes markedly significant 12 months after kidney transplantation (Figure 4(f)). There was no significant association between nitrated apoA-I or nitrated apoB with other known inflammatory and cardiovascular markers such as hs-CRP, MPO, and Cr. (data not shown).

## 4. Discussion

CVD in CKD is primarily driven by oxidative stress, vascular calcification, hypertension, inflammation, and accumulation of oxidized lipoproteins as well as HDL deficiency and dysfunction [39, 40]. Recent study showed that HDL function was impaired in heart transplant recipients but it was not related to cardiac allograph vasculopathy and CRP levels [41]. To date, there is insufficient information about HDL functionality in kidney transplant recipients. In the present report, we demonstrate for the first time that serum nitrated apoA-I is reduced after 12 months in kidney transplant recipients with good kidney function.

We examined associations of nitrated lipoproteins with serum levels of apoA-I and apoB in kidney transplant recipients. We found no significant relationship between nitrated apoA-I and levels of circulating apoA-I at baseline and at 3 months and 12 months after transplantation (Figures 4(a)–4(c)). This is not in agreement with our previous observation in CVD patients with low HDL [34]. This could be due to possible differences in lipoproteins and/or degree of nitration and patient's population from CKD and CVD. Surprisingly, there was a significant negative correlation between percent nitrated apoB and levels of serum apoB at baseline and at 3 months and 12 months after transplantation (Figures 4(d)–4(f)). The more the serum is enriched with apoB the lesser the degree of apoB nitration is. One plausible explanation to this unexpected observation could relate to differences in clearance of different forms of modified apoB-LDL particles. We speculate that nitrated apoB-LDL molecules would be cleared faster in the circulation than unmodified LDL resulting in lower amount of nitrated apoB. In fact, previous studies demonstrated that the accumulation of apoB-containing lipoproteins results primarily from decreased clearance rather than from increased synthesis [42]. LDL nitration has been shown to be associated with enhanced macrophages uptake when compared with oxidized LDL isolated from rheumatoid and osteoarthritis patients with CVD [43]. More studies are warranted to further elucidate the clearance of nitrated LDL particles by macrophages in CKD patients.

MPO is the main enzyme involved in chlorination and nitration of lipoproteins. Plasma MPO levels have been associated with coronary artery disease [44]. Cavusoglu et al. have shown that higher baseline MPO levels independently predict the occurrence of myocardial infraction within 2 years in patients with acute coronary syndrome [45]. High levels of nitrated HDL have been found in atherosclerotic lesions and plasma of CVD patients. In contrast, the majority of nitrated LDL mainly resides within atherosclerotic plaques [21, 46–48]. Use of drugs such as statins, beta-blockers, or ACE inhibitors has been shown to reduce MPO levels in patients with acute coronary syndrome but not in patients with stable coronary artery disease [49].

Interestingly, the decrease in apoA-I nitration 12 months after transplantation was associated with significant reduction of MPO activity (Table 1). It is not clear from our studies whether the reduced nitrated apolipoprotein A-I at 12 months is causally linked to reduced MPO activity. It is possible that reduction in serum concentration of MPO after successful kidney transplantation is due to attenuation of oxidant and inflammatory states induced by the uremia. In fact, recent study demonstrates that MPO deficiency ameliorates renal injury in the renal ablation model of CKD in mice [50]. Another study reports a negative correlation between MPO and urea and creatinine levels [51].

The evolution and severity of CKD have been shown to be associated with elevated oxidative stress [52]. Analysis of TBARS content in apoB-associated LDL/VLDL particles did not reveal any significant differences at 3 months and 12 months after transplantation compared to baseline (Table 1). This is quite different from the study by Vostálová et al. showing beneficial effect of successful kidney transplantation on the antioxidant status and lipid metabolism which resulted from both improved renal function and reduced cardiovascular complications [53]. One plausible explanation to this discrepancy could be related to differences in lipid status, patient selection, and protocol design between the two studies. In addition, the changes in lipid peroxidation products in CKD population are still debated [54–56].

In this study, a large proportion of subjects have high levels of serum apoB (>100 mg/dL; normal range ≤60 mg/dL) probably due to absence of lipid-lowering treatment in these patients and possible effect of immunosuppressive drugs. Unlike apoA-I, good kidney function did not affect degree of apoB nitration at 3 months and 12 months after transplantation (Figures 3(c) and 3(d)). These differences could be related to low levels of nitrated apoB in serum as compared to nitrated apoA-I. Alternatively, structural and molecular differences between lipoproteins may occur during progression of CKD, rendering modified LDL molecules in particular (oxidation, glycation, and carbamylation) less susceptible to nitration by MPO. Analysis of total population (all fourteen patients) with good graft function did not show any significant temporal changes in nitrated lipoproteins 1 month, 3 months, and 12 months after transplantation (Figure 2). This could be due to significant interindividual variations among patients in this small population. However, modest elevation of serum apoA-I levels was associated with significant reduction of nitrated apoA-I in kidney transplant recipients after only 12 months (Figure 3(b)). This is consistent with recent study reporting that changes of lipid profiles occur early and almost universally at 12 months after kidney transplantation [57].

One major limitation of our study is the small sample size due to strict patient's exclusion criteria setting. Nevertheless, none of our patients had received lipid-lowering therapy that could affect outcome of the study. Another limitation is lack of suitable normal control group (donors) for this particular setting protocol. These findings need to be confirmed in a large prospective study in order to validate the usefulness of nitrated apoA-I as independent predictor marker for CVD risk in CKD patients. More focused research is warranted to elucidate whether modified HDL in advanced CKD participates in very similar cellular processes of atherosclerosis such as foam cell formation, proliferation and migration of smooth muscle cells, and, most importantly, plaque destabilization.

## 5. Conclusion

In summary, we have demonstrated that patients with well-functioning kidney transplants had significant reduction in nitrated apoA-I-HDL 12 months after kidney transplantation without any major changes in nitrated apoB-LDL. Given the high cardiovascular burden of kidney transplant recipients, nitrated apoA-I may serve as valuable marker for population stratification and perhaps as a possible target for novel therapeutic strategies.

## Figures and Tables

**Table 1:** Clinical characteristics at baseline (before transplantation) and at 3 months and 12 months after transplantation.

Characteristics	Baseline (*n* = 14)^a^	3 months after transplantation (*n* = 12)	12 months after transplantation (*n* = 8)
Age at transplantation (years)	46.6 ± 12	45.1 ± 11.9	48.3 ± 15.8
Female (*n*)	6	6	4
BMI (kg/m^2^)	25.1 ± 3.26	24.7 ± 3.3	28.9 ± 4.1^*∗*^
Systolic BP (mmHg)	149.5 ± 13.6	128.3 ± 14.7^*∗∗∗*^	143.9 ± 18.1
Diastolic BP (mmHg)	86 ± 7.8	73.3 ± 11.8^*∗∗*^	81.5 ± 10.8
Blood urea nitrogen, BUN (mg/dL)	39.8 ± 20.8	22.9 ± 5.1^*∗*^	24.3 ± 8
Triglycerides (mg/dL)	121.30 [62.27–386.37]	135.60 [73.90–246.34]	103.40 [84.44–242.00]
Total cholesterol (mg/dL)	152.4 ± 36.5	187.3 ± 26.4^*∗*^	198.3 ± 81.7
Glycerol (mg/dL)	28.5 ± 23.1	29.4 ± 11.6	39.1 ± 33.8
Serum apoA-I (mg/dL)	76.76 [9.68–92.61]	71.99 [48.43–90.33]	79.19 [45.04–89.19]
Serum apoB (mg/dL)	129.13 [74.29–186.95]	115.84 [59.21–254.67]	158.20 [61.96–190.96]
Non-HDL cholesterol (LDL + VLDL, mg/dL)	107.1 ± 22.2	97.6 ± 27.3	118.2 ± 18.6
HDL cholesterol (mg/dL)	52.9 ± 16.7	65.8 ± 30.4	52.6 ± 17.7
TBARS (LDL + VLDL) (MDA nmoles/mL)	7.7 ± 5.7	7.5 ± 6.3	6.9 ± 3.0
Myeloperoxidase (mU/mL)	109.7 ± 27.0	110.4 ± 50.1	73.9 ± 53.0^*∗*^
Serum creatinine (mg/dL)	7.9 ± 2.5	1.2 ± 0.2^*∗∗∗*^	1.2 ± 0.3^*∗∗∗*^
eGFR (mL/min) (MDRD)	N/A	73.6 ± 14.5	74.9 ± 15.9
hs-CRP (mg/L) (median)^b^	<4	<4	<4
Hemoglobin (g/dL)	11.3 ± 2	12.5 ± 1.4	13.1 ± 1.4^*∗*^
Albumin (g/dL)	4.1 ± 0.4	4.3 ± 0.2	4.1 ± 0.1

Data are presented as means ± SD or medians [interquartile ranges]. Values from baseline (before transplantation) and 3 months and 12 months (after transplantation) were compared using nonparametric test (data were significant at  ^*∗*^ *p* < 0.05, ^*∗∗*^ *p* < 0.01, and ^*∗∗∗*^ *p* < 0.001).

^a^Fourteen patients had creatinine ≤1.5 at 3 or 12 months after transplantation; two of the 14 patients were not included at 3 months because creatinine was >1.5. The graft function of these two patients improved to creatinine of ≤1.5, so, together with 6 patients included at 3 months, they make up 8 patients at 12 months.

^b^50% of patients had values <4 (lower limit of detection for assay = 4 mg/L).

## Abbreviations

ABCA1: ATP-binding cassette transporter A1
ApoA-I: Apolipoprotein A-I
ApoB: Apolipoprotein B
BUN: Blood urea nitrogen
CKD: Chronic Kidney disease
Cr.: Creatinine
CRP: C-reactive protein
CVD: Cardiovascular disease
EGFR: Estimated glomerular filtration
ELISA: Enzyme linked immunosorbent assay
ESRD: End-stage renal disease
HDL: High-density lipoprotein
LDL: Low-density lipoprotein
MDA: Malondialdehyde
MDRD: Modification of diet in renal disease
MPO: Myeloperoxidase
VLDL: Very low-density lipoprotein
TBARS: Thiobarbituric acid reactive substances.
